# Premature ventricular contraction detection and estimation of daily burden by an insertable cardiac monitor

**DOI:** 10.1016/j.hroo.2025.01.004

**Published:** 2025-01-11

**Authors:** Kris Z. Siejko, Molly Kupfer, Abhijit Rajan, Keith Herrmann, Devi Nair

**Affiliations:** 1 Boston Scientific Corp, St. Paul, Minnesota; 2 St. Bernard’s Heart & Vascular, Jonesboro, Arkansas

**Keywords:** PVC burden, Ventricular ectopy, Insertable cardiac monitor, Remote monitoring, Ambulatory ECG, Cardiomyopathy

## Abstract

**Background:**

Premature ventricular contraction (PVC) burden is a clinically important metric in the context of PVC-induced cardiomyopathy and is commonly obtained via ambulatory electrocardiogram (ECG) monitoring.

**Objective:**

The purpose of this analysis is to characterize the performance of a novel PVC detection algorithm capable of identifying single PVCs and PVC sequences (couplets and triplets) for estimation of 24-hour PVC burden in an insertable cardiac monitor (ICM).

**Methods:**

Performance of the ICM algorithm for detecting PVCs was validated by replaying 748 patient-triggered ICM-recorded ECG episodes from 184 patients through the ICM device. To assess performance over longer ambulatory periods, a validated software model equivalent of the implemented ICM algorithm was evaluated against a 24-hour Holter dataset of 89 patients. The model also was used to evaluate performance on an established reference library from the Massachusetts Institute of Technology and Beth Israel Hospital (MIT-BIH Arrhythmia Database) as a basis of comparison with other published algorithms.

**Results:**

Beat-level validation on the ICM-stored episode dataset yielded a gross PVC sensitivity of 80.1% with a specificity of 99.7%. The correlation between 24-hour Holter burden and ICM algorithm PVC burden was *r* = 0.95. The sensitivity for identifying patients with PVC burdens ≥10% was 84%, with a patient-level positive predictive value (PPV) of 100%. Beat-level sensitivity of the PVC algorithm evaluated against the MIT-BIH dataset was 87.9% with a PPV of 96.4%.

**Conclusion:**

The ICM algorithm reliably detects PVCs with high sensitivity and specificity. Twenty-four-hour PVC burden measurements demonstrated a strong correlation with a gold standard 12-lead Holter and may provide utility for identifying patients at risk for worsening left ventricular function.

## Graphical abstract

**Figure:**
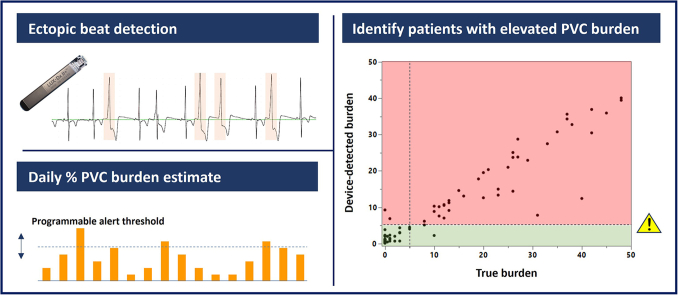

**Key Findings**

▪ A novel PVC detection algorithm, capable of detecting single PVCs and PVC sequences, was developed for an insertable cardiac monitor.
▪ The ICM algorithm demonstrates high sensitivity for detecting ectopic beats and identifying patients with elevated PVC burden.
▪ ICM-detected burden demonstrates strong correlation with a gold-standard 12-lead Holter monitor, suggesting potential utility in screening for patients at risk of left ventricular dysfunction.

## Introduction

Cardiac arrhythmias, specifically premature ventricular contractions (PVCs), have long intrigued clinicians and researchers because of their prevalence and potential impact on cardiac function. PVCs represent a common electrocardiographic finding in both symptomatic and asymptomatic individuals. Although PVCs are relatively common and often benign, when they occur frequently or are associated with specific risk factors, they can pose a significant burden on cardiovascular health. Numerous clinical studies have reported an association between frequent PVCs and an increased risk of cardiomyopathy, with impaired left ventricular function and structural changes observed in affected individuals.^1^, ^2^, ^3^, ^4^

PVC burden is a clinically useful metric defined as the percentage of PVC beats relative to all beats measured over 24 hours. The relationship between PVC burden and cardiomyopathy is a multifaceted and evolving field that warrants close examination. Although there is no consensus on what constitutes a clinically actionable PVC burden threshold, it is generally accepted within medical guidelines that a burden of 10% or higher can lead to development of left ventricular dysfunction,^5^^,^^6^ although instances of cardiomyopathy have been reported with burdens as low as approximately 5%.^7^

PVCs may be concentrated in periods of high density or spread uniformly throughout the day; thus, a patient’s electrocardiogram (ECG) must be monitored continuously to yield an accurate daily PVC burden measurement. Ambulatory Holter monitoring is conventionally used to measure 24-hour PVC burden. However, PVC burden has recently been demonstrated to vary substantially from day to day^8^; thus, an extended multi-day monitoring period is advantageous for a more representative assessment of the burden. With the advent of insertable cardiac monitors (ICMs), it is now feasible to assess daily PVC burden over an extended monitoring period without cutaneous wires or patches.^9^, ^10^, ^11^ The continuous beat-to-beat ECG processing required for PVC burden necessitates that PVC detection be performed within the ICM processor.

In this analysis, a novel PVC detection algorithm was evaluated. The algorithm, which is implemented in the LUX-Dx II and the LUX-Dx II+ ICM (Boston Scientific, St. Paul, Minnesota), quantifies 24-hour PVC burden, which includes both single PVCs, including bigeminy, and sequences (couplets, triplets). The algorithm has a configurable minimum burden threshold starting at 5% with an associated alert. Beat-level PVC detection performance was evaluated on the ICM device by replaying patient-triggered ECG recordings from the predecessor LUX-Dx ICM. The ability of the algorithm to accurately estimate 24-hour PVC burden was assessed in silico against real-world Holter data via a validated software model equivalent of the PVC detection algorithm. Finally, the software model was used to compare performance of the ICM algorithm directly with other published PVC algorithms, using an established benchmark database.

## Methods

### Algorithm description

The ICM PVC Burden algorithm is more accurately a *ventricular ectopic beat* detector; PVC beats are not strictly required to be premature. Couplets, triplets, and some fusion beats and escape beats are included in the PVC tally used to compute burden percentage. Herein all forms of detected ventricular ectopy are referred to as PVCs.

**Figure 1:**
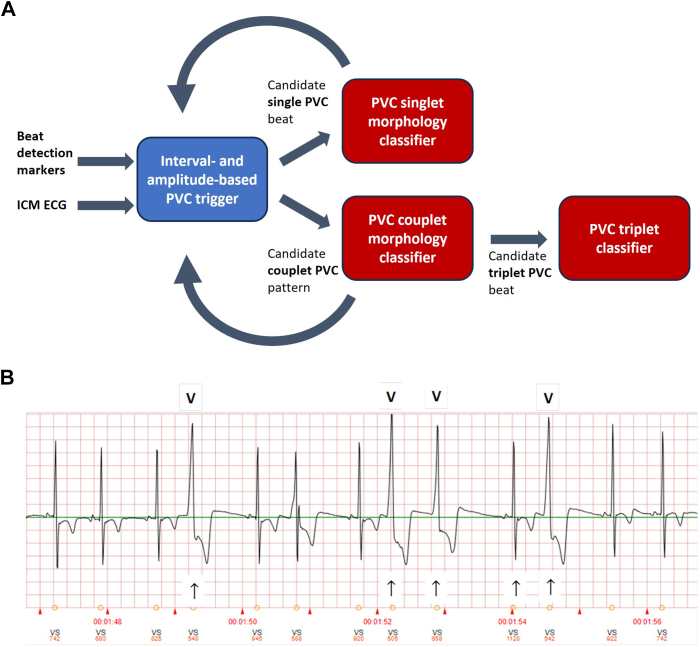
**A:** A 2-stage PVC algorithm efficiently uses ICM processor resources via a PVC candidate trigger stage followed by a morphology-based classifier on the reduced set of beats. Different beat classifier configurations are used depending on PVC sequence type. **B:** Example of ICM-stored ECG signal segment overlayed with PVC algorithm results. *V* denotes ICM algorithm PVC detections, *arrows* are interim algorithm-triggered PVC candidates. ECG = electrocardiogram; ICM = insertable cardiac monitor; PVC = premature ventricular contraction.

The PVC detection algorithm has 2 stages: a trigger stage, which efficiently considers every detected beat to identify interval and amplitude patterns of potential PVCs, followed by a morphology stage, which performs a more comprehensive and computationally intensive assessment to declare each triggered beat as either a PVC or non-PVC (Figure 1A). Intervals containing noise markers are excluded during the trigger stage and help to reduce PVC false positives. The detected PVC beats are summed and normalized by the total number of ICM beat detections over 24 hours to arrive at a daily PVC burden percentage. Figure 1B provides an example of the output of the stages of the algorithm for an ICM-recorded ECG segment.

The morphology stage uses a feature-based logistic regression classifier. A machine learning approach was used to train the classifier using a large volume of beat-adjudicated surface and ICM ECG data, through which the classifier weights were determined. The QRS-descriptive features used by the classifier make extensive use of Correlation Waveform Analysis (CWA) and dynamic CWA (DCWA) template matching techniques; these are also used by Boston Scientific subcutaneous implantable cardioverter-defibrillators.^12^^,^^13^ A CWA template formation step, representing an average normal sinus rhythm (NSR) QRS complex, is attempted every 2 hours by the ICM. Amplitude-based metrics (eg, peak amplitude ratios of surrounding beats, slew rates) are used as well. Separate classifiers were trained for single, couplet, and triplet PVC sequences, in which a unique combination of features and coefficients were determined for each type of PVC temporal sequence.

### Datasets

Algorithm performance was evaluated on independent datasets from 3 different sources: (1) patient-triggered episode recordings from ambulatory LUX-Dx ICM patients; (2) 24-hour Holter recordings from Mayo Clinic patients with noted presence of either PVCs or arrhythmias; and (3) a publicly available dataset from the Massachusetts Institue of Technology and Beth Israel Hospital (MIT-BIH Arrhythmia Database),^14^^,^^15^ consisting of 22 patients. The datasets are summarized in Table 1. All data were de-identified and accessed through retrospective data review, so this analysis does not constitute human subject research as defined by the US Department of Health and Human Services Office for Human Research Protections 45 CFR part 46. LUX-Dx data was obtained from the LATITUDE Clarity data management system with approval from Boston Scientific’s Data Governance Board and the permission of the data owners, and in accordance with the US Health Insurance Portability and Accountability Act. Data from the Mayo Clinic was de-identified in accordance with applicable laws and regulations, and de-identified data from the MIT-BIH Arrhythmia Database are publicly available.

**Table 1:** Summary of datasets

Dataset	No. of patients	Total duration	Average PVC burden	N PVCs	% PVC couplets and triplets
ICM patient-triggered episodes	184	94 hours	0.84%	4212	11.6%
24-h Holter	89	85.6 days	11.6%	1.05M	11.2%
MIT-BIH arrhythmia	22	11 hours	6.5%	3321	9.6%

ICM = insertable cardiac monitor; MIT-BIH = Massachusetts Institute of Technology-Beth Israel Hospital; PVC = premature ventricular contraction.

The ICM episode set was used to validate the performance of the detection algorithm for detecting PVCs and estimating burden over short periods, as well as to validate an equivalent software model of the ICM algorithm. The 24-hour Holter data were used to assess the accuracy of the ICM algorithm in estimating PVC burden over a full 24-hour period relative to a commercial Holter PVC detection system. Finally, the MIT-BIH Arrhythmia Database was used as a standardized ECG dataset to evaluate the new algorithm against state-of-the-art PVC detection algorithms. All datasets contained a relatively high percentage of PVCs from sequences (couplets and triplets).

#### ICM-stored episode dataset

The patient-triggered ICM-recorded data were obtained from a randomly selected sequestered subset from the first 700 LUX-Dx US patient implants via the LATITUDE Clarity Data Management System. The validation set represents 72 different US clinical sites. To ensure that the dataset was not overrepresented by a few patients with a large number of episodes, a limit of 8 consecutive episodes per patient was imposed; because each patient-triggered recording is approximately 7.5 minutes long, this limit corresponds to approximately 1-hour maximum duration per patient. Episodes with excessive and persistent noise artifact with mean R-wave amplitudes <100 μV were excluded, because these amplitudes fall 50% or more below the minimum recommended level of 200 μV. Real-time replay of prerecorded ECG data into the ICM device hardware was accomplished using a special test station with a streaming digital to analog converter, which applies an analog (voltage) waveform to the ICM electrodes. A 30-second segment of NSR is appended to the beginning of each episode segment to facilitate automatic NSR template formation used by the PVC algorithm. Inverse digital prefilters were applied to prevent double-filtering effects of replay.

The episode waveforms were prospectively adjudicated for PVCs by trained clinical experts. ICM episode beat detection timing markers were provided; adjudicators marked only those beat detections deemed PVCs or ambiguous. Positions of PVCs that were undersensed were manually added. Each file was first fully adjudicated by a single reviewer, and a second reviewer only inspected PVC files containing ambiguous PVC markers as designated by the first reviewer. Additional expert reviewers were consulted as needed for files with many ambiguous beats. Beats that remained ambiguous after the second review were excluded from analysis as prescribed by Association for the Advancement of Medical Instrumentation standard EC57.^16^

#### 24-Hour Holter dataset

Twenty-four-hour Holter data and accompanying reports were procured from the Mayo Clinic (Rochester, MN). The recordings were selected by Mayo clinicians from a pool of preexisting Holter data with noted arrhythmias, PVCs, premature atrial complexes (PACs), and pauses. Data were collected via the GE Spacelabs (Snoqualmie, WA) Lifecard CF 12-lead Holter system, and the Pathfinder SL analysis software was used to process and analyze the Holter data and automatically generate the summary reports. The resulting PVC burden for each patient was determined directly from the Holter report, using the ratio of ventricular ectopic beats to the total number of beats.

One hundred Holter patients were sequestered for independent testing, but 11 patients with >1% pacing were excluded, because wide pace-evoked responses are susceptible to misclassification as PVCs. The final dataset thus consisted of a total of 85.6 patient-days of data from 89 patients. The 24-Hour Holter dataset characteristics according to the Pathfinder reports are shown in Table 2. The composite precordial lead V2-V3 signal from Holter was used to approximate the ICM vector, as previously described.^17^ A validated high-fidelity MATLAB software model (Mathworks, Natick, MA) of the PVC detection was employed to facilitate faster than real-time processing of the 24-hour Holter dataset.

**Table 2:** Characteristics of 24-hour Holter data used for PVC burden algorithm validation

No. of patients	89
Total duration	85.6 patient-days
Mean reported burden	11.6%
Median reported burden	3.4%
Mean no. PVCs/patient	10,278
Patients with PVC burden >10%, N (%)	38 (43%)
Patients with PVC burden < 5%, N (%)	46 (52%)
Mean single PVC burden	10.3%
Mean recording duration	23.5 hours
Patients with PVC couplets, N (%)	67 (75%)
PVC sequence burden, mean, max.	1.3%, 15.7%
Mean PAC burden	0.9 % (in 12 patients)
Patients with PAC burden > 10%, N (%)	2 (2%)
Patients with atrial fibrillation, N (%)	13 (15%)

PAC = premature atrial contraction; PVC = premature ventricular contraction.

#### MIT-BIH arrythmia dataset

The publicly available MIT-BIH Arrhythmia Database has been used extensively by PVC algorithm developers as a benchmark for performance evaluation.^18^ There are 44 patients total, partitioned into training (DS1) and testing (DS2) sets of 22 patients each, with 30-minute 2-lead ECG recordings. This dataset is independent because it was not used for training or algorithm development. Only a single lead (modified lead II) of DS2 was used in this evaluation, because the ICM PVC algorithm is strictly a single-vector algorithm. Beat-level annotations are provided, including normal (N), supraventricular ectopic beats (S), PVCs (V), ventricular fusion beats (F), and ambiguous beats (Q). These provided markers are also commonly used as the beat detection fiducial points in published reports, because R-wave sensing is often cited as being out-of-scope for PVC beat classification algorithm development efforts. Thus, for direct and fair comparison against benchmark algorithm performance, the validated software model of the PVC detection algorithm was configured to bypass the filtering and beat-sensing stages to instead use the provided markers as beat detection locations.

For analysis of this dataset, only fusion (F) beats were considered ambiguous and excluded from sensitivity and positive predictive value (PPV) metrics, because fusion (F) beats were rare and typically had long coupling intervals that are often missed by the trigger stage. Moreover, reported benchmarks^18^ most often do not include fusion beats with the ventricular ectopic beat class.

### Performance metrics and statistical methods

PVC performance at the beat-level was characterized by gross sensitivity, specificity, and PPV. Patient-average metrics and Generalized Estimating Equation (GEE) methods were also used for the ICM dataset to calculate adjusted means and their 95% confidence intervals for each of the metrics and correct for the inherent correlations between multiple PVC observations per patient. A binomial link function and exchangeable correlation structure were used for the GEE method. A ±150 ms tolerance window relative to adjudicated PVC locations was used in PVC detection scoring. The specificity definition was modified to be normalized by detected beats and not by adjudicated normal beats, because beat detection performance of the LUX-Dx ICM has been previously validated. Burden estimation accuracy was assessed using the Pearsons correlation coefficient (*r*-value) between the ICM estimated PVC burden value and the reference burden value derived from adjudicated beat tallies, or in the case of the 24-hour dataset, the Holter-reported burden.

## Results

### Algorithm performance for the ICM episode dataset

Beat-level PVC detection performance of the PVC algorithm was assessed on the ICM episode dataset played through the LUX-Dx II+ test station as analog signals. Results are presented in Table 3. Because stored ICM episodes are undersampled and band limited to 40 Hz, the myopotential noise rejection mechanism (noise marker generation) is ineffective for replayed ICM episodes. The validated software model, however, enables direct substitution of the original field-detected beat detection locations and any noise markers in place of the modeled beat detection stages. When doing so, there is a small reduction in false PVC detections and gross PPV improvement (Table 3). The ICM device replay and validated software model results taken together provide a more accurate representation of algorithm performance.

**Table 3:** Beat-level PVC detection performance evaluated on a LUX-Dx II+ ICM device and validated software model equivalent.

Evaluation Method	Metric	Gross performance	Patient average	GEE and confidence intervals
ICM device replay	PVC sensitivity	**80.1%**	72.2%	72.9% (67.1%–78.1%)
	PVC specificity	**99.7%**	99.6%	99.6% (99.5%–99.7%)
	PVC PPV	**71.4%**	43.9%	44.1% (37.5%–50.8%)
Software model (using original ICM beat detection and noise markers)	PVC sensitivity	**81.0%**	73.3%	73.7% (67.7%–79.0%)
	PVC specificity	**99.8%**	99.7%	99.7% (99.5%–99.8%)
	PVC PPV	**74.4%**	44.3%	44.5% (38.0%–51.2%)

ICM = insertable cardiac monitor; PVC = premature ventricular contraction; GEE = generalized estimating equation.

Detected PVC sequences constituted 6.1% of all ICM PVC detection events. Twenty patients (11%) had adjudicated PVC couplets or triplets, accounting for 28% of their total true PVC tally. Eight (8) patients from this subgroup had true PVC burdens of ≥5%, where detected PVC sequences constituted on average 16% of all ICM PVC counts (ranging from 2% to 57%), or a mean of 2.3 percentage points out of the total ICM-estimated percent PVC burden (range, 0.1–6.8 percentage points [ppt]). For detection of single PVCs only, where adjudicated PVC sequences were removed from consideration, the gross sensitivity and PPV for device-replayed episodes were 84.6% and 70.5%, respectively; using the model (with noise markers), they were 85.1% and 73.4%.

**Figure 2:**
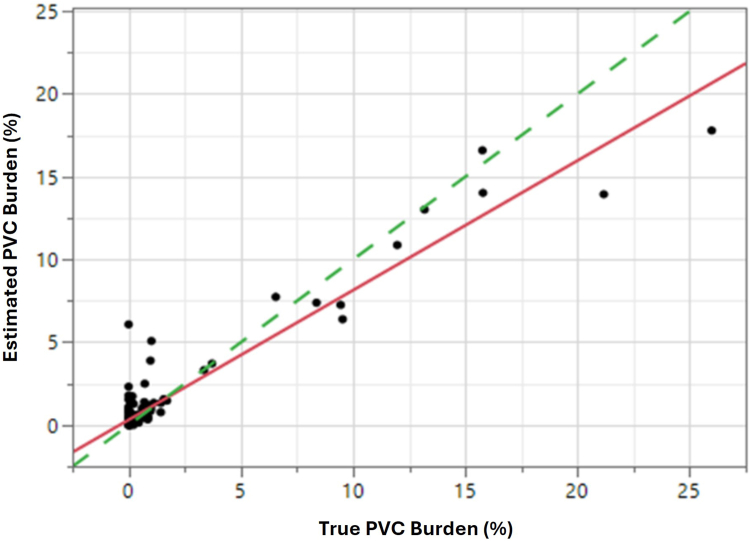
PVC burden performance of ICM episodes played back through LUX-Dx II+ ICM device versus adjudicated (true) PVC burden. Both linear fit and identity reference lines (dashed) are shown. ICM = insertable cardiac monitor; PVC = premature ventricular contraction.

PVC burden metric values were also calculated for each patient (mean ECG duration per patient = 0.51 hours). A scatterplot of ICM replay vs true (adjudicated) PVC burden (*r* = 0.95; *P* ≤ .0001) is shown in Figure 2. Patient-level performance for detection of elevated PVC burdens using a 10% threshold was 100% for sensitivity, specificity, and PPV in this dataset.

### ICM algorithm evaluation on 24-hour Holter dataset

**Figure 3:**
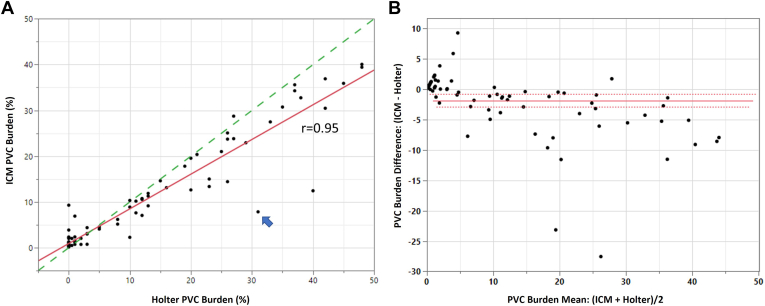
**A:** Performance of ICM PVC algorithm (via validated model) on the Holter 24-hour Holter dataset. The *unity line (dashed)* is also shown for comparison. The *arrow* identifies an example of a patient with underestimation of PVC burden caused by PVCs with narrow QRS projections that are mistaken for PACs (see Figure 4 for additional analysis). **B:** Bland-Altman plot comparison of ICM and Holter PVC burden estimates. ICM = insertable cardiac monitor; PVC = premature ventricular contraction.

The validated model was used to assess longer-term ambulatory performance of the PVC algorithm using the Holter dataset. Figure 3A shows the 24-hour burden estimation of the model for each patient vs the true burden according to the Pathfinder Holter reports. The linear correlation was *r* = 0.95 (*P* < .0001). Figure 3B is the corresponding Bland-Altman plot showing the relative bias of the ICM burden estimate. Overall, the ICM underestimated the Holter PVC burden by a mean of 1.83 ppt., with a tendency to overestimate low burdens less than 5%. Because beat adjudications were not available, gross sensitivity could not be calculated; however, a surrogate ratio of the total number of ICM PVC detections to the total number of Holter PVC detections was 88%. Overall, 5.7% of ICM PVC detections were couplets and triplets. Forty-three (43) patients had Holter burden ≥ 5%; of those patients, PVC sequences accounted for a mean of 12% of the total Holter PVC count (range, 0%–94%); the corresponding ICM-detected burden sequences accounted for a mean of 1.0 ppt of the total burden percentage (0 to 5 ppt). Thirty-eight patients had a true PVC burden above 10%; use of a corresponding 10% ICM threshold yielded 6 missed patients with no false positives (sensitivity = 84%, specificity = 100%, PPV = 100%).

### ICM algorithm comparison with state-of-the-art

Table 4 summarizes the performance of the PVC algorithm on the standardized MIT-BIH dataset compared against other representative published performance benchmarks, many of which are more computationally intensive than what could be implemented in an ICM processor. The referenced studies are directly comparable to the ICM results in that none of the datasets were used for algorithm training, and most used a single lead. Moreover, studies with intrapatient partitioning of training and testing subsets also were not considered. The ICM algorithm demonstrated a high PPV with comparable sensitivity and F1 score (the harmonic mean of sensitivity and PPV) to the comparator algorithms. Detected PVC sequence beats (couplets, triplets) accounted for 5.5 percentage points of the gross sensitivity. In addition, burden-level analysis on the MIT-BIH dataset showed that the ICM algorithm had a linear correlation of *r*^*2*^ = 0.988 vs the true PVC burden, with a root mean square error of 0.95%.

**Table 4:** Performance of the ICM PVC algorithm on the MIT-BIH Arrhythmia Database. Other published PVC algorithm results are provided for direct comparison.

Algorithm	Sensitivity (%)	PPV (%)	F1	Classifier	Leads used
**ICM PVC algorithm using MIT-BIH beat marker locations**	**87.9**	**96.4**	**91.9**	**Logistic regression**	**II**
De Chazal et al, 2004^19^	77.7	81.9	79.7	Weighted LDs	II + V1’
Raj et al, 2016^20^	87.5	65.4	74.9	SVM	II
Al Rahhal et al, 2016^21^	91.0	79.5	84.9	Deep NN	II
Herry et al, 2017^22^	77.5	79.1	78.3	SVM	II
Huang et al, 2014^23^	93.9	90.9	92.4	SVM ensembles	II
Alfaras et al, 2019^18^	92.7	95.7	94.2	NN ensembles	II
Sultan et al, 2017^24^	95.4	94.1	94.7	Decision Trees	II
Teijeiro et al, 2018^25^	94.6	96.8	95.7	Rule-based	II+V1’

LD = linear discriminator; NN = neural network; PVC = premature ventricular contraction; SVM = support vector machine.

## Discussion

Three independent datasets were used to provide a comprehensive performance characterization of the LUX-Dx II/II+ ICM’s new PVC burden monitoring capability. The novel algorithm demonstrated comparable sensitivity and specificity for beat-level PVC detection and 24-hour PVC burden in comparison with gold-standard Holter reports and state-of-the-art algorithms.

### Detection of ventricular ectopic beats

The PVC algorithm demonstrated high sensitivity, specificity, and PPV for detection of PVCs in the ICM dataset. Most missed PVCs detections were attributable to missed triggers and undersensing of PVCs of very low amplitude, accounting for 10 ppt of the total sensitivity loss. The most common source of false-positive PVC detections was conducted PACs with aberrancy, with less common sources being motion artifacts and abrupt changes in NSR beat morphology (eg, due to posture changes). When evaluated on the MIT-BIH dataset, the ICM PVC algorithm performance compared favorably with other published algorithms (Table 4), most of which were considerably more computationally intensive and not suited for ICM processor implementation.^18^^,^^24^ It is also notable that the ICM algorithm’s PPV was superior to all comparators presented in Table 4 except for one algorithm that uses a 2-vector approach.^25^ The strong performance of the ICM algorithm on the MIT-BIH dataset (also relative to the ICM episode set) suggests its performance is influenced more by signal content and high PVC prevalence and less so by any limitations of the algorithm design. It also demonstrates robustness to electrode and lead placement.

### Estimation of PVC burden

**Figure 4:**
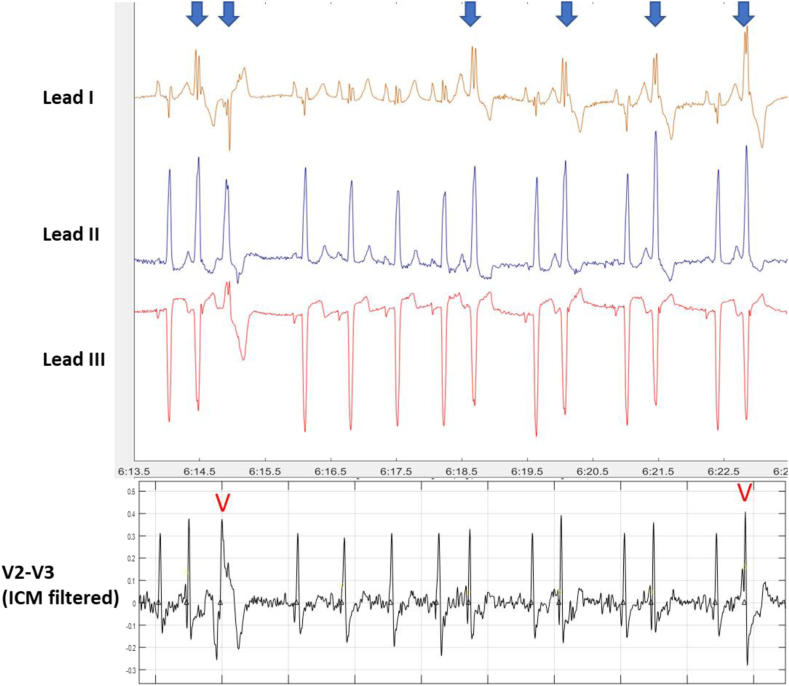
Example of underestimation of PVC burden (for the patient denoted by the *arrow* in Figure 3) caused by narrow projection of PVCs onto ICM vector. The *blue arrows* above indicate true PVCs clearly apparent in Lead I from Holter monitoring. The *bottom trace* shows the ICM vector simulated by V2-V3 and filtered by the ICM device for PVC detection. The ICM detected the 2^nd^ beat of a couplet and the 4^th^ bigeminal PVC (V symbols), but others were missed because of high similarity to NSR beats. ICM = insertable cardiac monitor; NSR = normal sinus rhythm; PVC = premature ventricular contraction.

There was a strong correlation between ICM algorithm-estimated PVC burden and Holter-estimated 24-hour PVC burden. Overestimation of PVC burden was predominantly attributable to conducted PACs with aberrancy; these appear as PVC-like beats with QRS morphologies that differ substantially from the NSR beats. Cases of significant underestimation of PVC burden occurred because of PVCs with narrow QRS projections in the ICM vector. Figure 4 shows an example of PVC complex narrowing for the typical ICM sensing vector. Patient-level PVC burden performance on the ICM dataset represented shorter periods (≤1 hour per patient) but showed a similarly strong correlation with true beat-adjudicated PVC burdens. Although the beat-level PPV performance of Table 3 suggests a surplus of false-positive PVCs, these were primarily scattered among the large proportion of patients with very low burdens (well below 5%) and thus had little practical effect on individual PVC burden estimates.

### Detection of PVC sequences

The ability of this PVC algorithm to detect couplets and triplets differentiates it from other ICM-based PVC algorithms. Previously published analyses of ICM PVC detection algorithms^9^^,^^10^ have ignored sequences, likely because of the inherent difficulty for a real-time ICM algorithm to identify them reliably and because they typically constitute only a small percentage of total PVC beats. In this study, PVC sequences indeed represented a small percentage of the overall PVCs observed across all patients evaluated. However, in a subset of patients, PVC sequences constituted a large portion of their overall PVC counts. In such cases, failure to detect couplets and triplets would lead to a significant underestimation of the patient’s true PVC burden.

Beat-level sensitivity for couplet and triplet beats is expected to be lower than for single PVCs. This is reflected in the comparison of performance metrics for single PVC vs overall PVC beat detection, in which a slightly higher sensitivity for single PVCs was observed. However, detection of couplet and triplet beats raises the overall PPV and yields a more accurate estimation of PVC burden, which is especially important in those patients who experience a higher burden of sequence PVC beats.

### Clinical use and alert thresholds

Evaluation of the PVC algorithm against both the patient-triggered ICM episode dataset and the 24-hour Holter dataset indicated a general tendency to underestimate true elevated PVC burdens above 5%. Because the LUX-Dx II/II+ ICMs have a configurable PVC burden alert threshold, the effects of threshold selection on performance should be considered. For example, on the Holter dataset, selecting a threshold of 10%, which is often considered a minimum burden level for risk of PVC-induced cardiomyopathy,^5^^,^^6^ yields a patient-level sensitivity of 84%, with a corresponding PPV of 100%. Lowering the threshold to 5% would compensate for burden underestimation and increase patient-level sensitivity to 93% (for true burdens ≥5%) with a small reduction in patient-level PPV to 95%. (For the ICM dataset, lowering the threshold from 10% to 5% would maintain 100% patient-level sensitivity but decrease the PPV from 100% to 83%.) Physicians may configure the device’s alert threshold based on their approach to managing patients and the burden level they consider to be clinically actionable while taking into consideration the potential for burden underestimation.

Follow-up with a conventional Holter is a sound approach for patients for whom the ICM detects elevated PVC burdens, because no ECG strips are provided specifically for the PVC burden feature. This action can rule out PACs and other confounders, corroborate the ICM-estimated PVC burden, and assess the presence of multiple PVC morphologies. Alternatively, use of the patient-triggered recording feature of the ICM can be considered for capture of additional ECG segments to assess PVC morphologies, particularly for patients experiencing symptomatic palpitations. Once a true PVC burden baseline is established and appropriate clinical action is taken at the discretion of the physician, the ICM-reported PVC burden trend can continue to monitor long-term trends and changes in burden. Given the variety of ways different physicians approach risk management in patients with PVCs, the PVC burden monitoring and alert feature can be tailored for different strategies or patient scenarios.

### Limitations

This analysis represents a simulation of performance of the PVC algorithm using prerecorded ECG data, which presents some technical limitations as described in the Methods. However, data used for validation of the algorithm included real-world ICM signals obtained in vivo in addition to data from patients monitored via 24-hour Holter. The datasets used in this analysis represent a mixture of sinus rhythm and rhythm abnormalities that may be expected during monitoring, so the performance metrics presented here may not be applicable to select populations with higher burdens of atrial arrhythmias or PACs or to patients with conduction disorders.

## Conclusion

The LUX-Dx II/II+ ICM PVC burden feature provides accurate detection of ventricular ectopic beats and estimation of daily PVC burden and is a valuable addition to the suite of existing ICM arrhythmia detection capabilities. Despite typical confounders associated with use of a single ECG vector, the ICM provides high sensitivity in screening of patients with elevated PVC burdens. Further investigation is warranted into the potential utility of this tool to risk-stratify patients for PVC-induced cardiomyopathy or long-term ectopy monitoring.
